# Analysis of Hydrocephalus after Decompressive Craniectomy for Traumatic Brain Injury

**DOI:** 10.12669/pjms.41.8.11324

**Published:** 2025-08

**Authors:** Zhiyu Gao, Guanghui Zhang, Chongfu Xu, Zipeng Zhu, Haitao Jiang

**Affiliations:** 1 Zhiyu Gao Department of Neurosurgery, Liaocheng People’s Hospital, Liaocheng, 252000, Shandong, P.R. China; 2 Guanghui Zhang Department of Neurosurgery, Liaocheng People’s Hospital, Liaocheng, 252000, Shandong, P.R. China; 3 Chongfu Xu Department of Neurosurgery, Liaocheng People’s Hospital, Liaocheng, 252000, Shandong, P.R. China; 4 Zipeng Zhu Department of Neurosurgery, Liaocheng People’s Hospital, Liaocheng, 252000, Shandong, P.R. China; 5 Haitao Jiang Department of Neurosurgery, Liaocheng People’s Hospital, Liaocheng, 252000, Shandong, P.R. China

**Keywords:** Traumatic brain injury, Post-traumatic hydrocephalus, Subdural hygroma, Interhemispheric hygroma

## Abstract

**Objective:**

To explore the risk factors of post-traumatic hydrocephalus (PTH) in patients with traumatic brain injury (TBI) decompressive craniectomy (DC).

**Methods:**

This was a retrospective study. The objects of study were 92 TBI patients who met the eligibility criteria for routine DC treatment in the Department of Neurosurgery of Liaocheng People’s Hospital from January 2022 to December 2023. Furthermore, risk factors of PTH were determined by univariate and multivariate Logistic regression analysis.

**Results:**

According to the results of univariate analysis, there was correlation of PTH formation with DC grading, midline shift, operation time, intraoperative blood loss, tracheotomy, subdural hygroma, interhemispheric hygroma, ventricular dilatation, postoperative large-area cerebral ischemia and infarction. Furthermore, multivariate Logistics regression analysis revealed that the occurrence of subdural hygroma (OR 3.392 [95% CI 1.259-9.137]; p=0.016) was an independent prognostic factor of PTH. In addition, compared with non-operation group (conservative group, n=12) with lumbar puncture and/or lumbar cistern drainage and/or lateral external ventricular drain (LEVD), the curative effect was significantly better in the operation group with cranioplasty (CP) and/or ventriculo-peritoneal shunt (VPS) (p=0.029).

**Conclusion:**

The occurrence of subdural hygroma is an independent risk factor for hydrocephalus formation. For the cases of PTH combined with TBI, it is recommended to carry out individualized precision treatment according to the specific situation to control the malignant progression of hydrocephalus.

## INTRODUCTION

Decompressive craniectomy (DC) is a last treatment option of refractory post-traumatic intracranial hypertension in traumatic brain injury (TBI), with multiple postoperative complications. Post-traumatic hydrocephalus (PTH) is one of the most common complications.^1^,^2^ The clinical manifestations of PTH are progressive enlargement of ventricle and continuous no improvement or aggravation of nerve dysfunction.^2^

The incidence of PTH in TBI patients after DC varies from 6.3% to 54.0% due to different diagnostic criteria and interventions.^2^,^3^ PTH may occur within weeks, months or even a year after DC, mainly around one month after operation. The occurrence of PTH commonly causes aggravation in the illness condition of TBI patients, leading to poor prognosis, and even death in severe cases.^2^-^5^ According to multiple previous reports,^6^ factors related to the formation of PTH include age, osteotomy area, subarachnoid hemorrhage, intraventricular hemorrhage, Glasgow Coma Scale (GCS) score at admission, intracranial infection, subdural hygroma, interhemispheric hygroma, vertical distance (<25 mm) from the edge of bone flap to the sagittal suture, and delayed cranioplasty (CP). However, considering different evaluation and inclusion criteria of PTH in different Research Centers, there is no consistent understanding of the risk factors and mechanism of PTH in TBI patients after DC.

In this study, a retrospective analysis was performed by involving 92 TBI patients with PTH after DC. It is expected to provide a new reference for the occurrence and development, early diagnosis, risk prediction and precise treatment of PTH.

## METHODS

A retrospective analysis was performed by collecting the clinical data of 92 TBI patients who met the eligibility criteria for routine DC treatment in the Department of Neurosurgery of Liaocheng People’s Hospital from January 2022 to December 2023.

### Ethical approval:

This study was approved by the Institutional Ethics Committee of the Liaocheng People’s Hospital (No.:QFYLL2023010, March 2023), and written informed consent was obtained from all participants.

### Inclusion criteria:

- Surgical indications were based on the latest literature.
- TBI Management Guidelines issued by the American Brain Trauma Foundation.^7^

### Exclusion criteria:

- Severe compound injury.
- Severe coagulation dysfunction.
- Oculomotor nerve injury.
- Hypoxemia (PaO2<70 mmHg).
- Hypotension (systolic pressure <90 mmHg).
- Death within seven days after DC.^8^
- PTH generally did not have enough time to form within one week after DC, and all these patients were excluded from the study.

### Diagnostic criteria of PTH:

**Fig.1:**
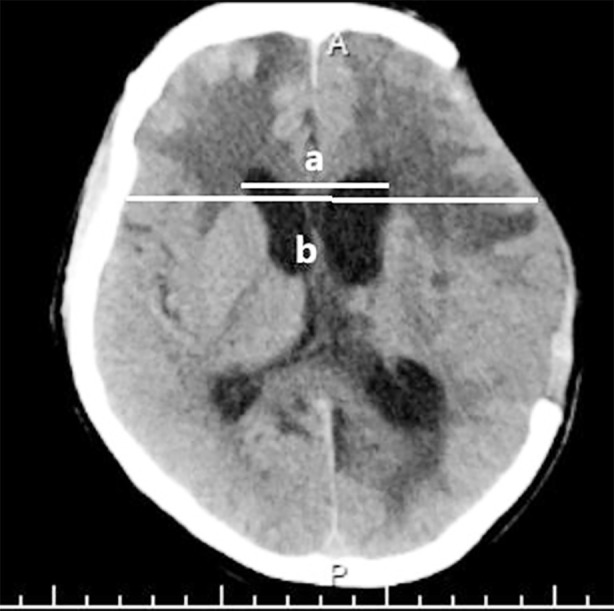
Axial cranial CT scan after left routine frontal-temporal-parietal decompressive craniectomy to measure the modified frontal angle (a/b).

- Clinical manifestation: consistent coma after DC, or repeated aggravation of consciousness disorder after improvement and deterioration of neurological function, or continuous swelling and gradual increase of the tension of cranial decompression window.
- Imaging features: expansion of ventricles and progressive aggravation of interstitial edema in the dynamic observation of cranial CT, external hydrocephalus or decrease of the thickness of brain tissue after increase, etc., and improved frontal angle index >0.33^9^(the maximum width of the frontal angle divided by the bi-cortical distance in the same plane, Fig.1), etc.;
- Test of draining with lumbar puncture (TAP test)^10^: positive TAP test, repeated if necessary, to increase sensitivity.

Two trained resident physicians in the Department of Neurosurgery counted the epidemiological data, clinical and imaging data, operation conditions and postoperative complications of the enrolled patients.

### Curative effect assessment:

Follow-up was performed for at least six months. The follow-up work of all patients was completed by the same group of surgeons. According to GOSE scores,^11^ the prognosis was divided into unfavorable prognosis (GOSE 1-4) and favorable prognosis (GOSE 5-8).

### Data analysis:

SPSS statistical software (version 22.0, IBM SPSS statistics) was used for data analysis and processing. The significance level α was set to 0.05 and the confidence interval was 95%. The measurement data was represented by mean ± standard deviation. The normal distribution data was subject to student *t* test, and the non-normal distribution data was evaluated by Mann Whitney U-test. Meanwhile, the categorical variables were expressed by rate, and the differences between groups were compared by chi-square test or Fisher’s exact test. P<0.05 meant that the difference was statistically significant for all statistical analysis.

## RESULTS

The clinical characteristics of 92 patients with TBI who underwent DC are shown in Table-I. Table-II describes the relationship of subdural hygroma and interhemispheric hygroma with hydrocephalus in 92 patients with TBI undergoing DC.

**Table-I:** The clinical characteristics of 92 patients with TBI who underwent DC.

Variables	Total numbers*	Hydrocephalus*		P value
		With	Without	
Number of patients	92	22	70	
Gender (%)				0.486
Male	56(60.9%)	12(54.5%)	44(62.9%)	
Female	36(39.1%)	10(45.5%)	26(37.1%)	
Average age (years)	47.03 ± 14.27	46.05 ± 12.00	47.34 ± 14.98	0.712
≥50	40(43.5%)	8(36.4%)	32(45.7%)	
<50	52(56.5%)	14(63.6%)	38(54.3%)	
Preoperative GCS score	5.47 ± 2.05	5.50 ± 1.95	5.46 ± 2.09	0.777
Midline shift (mm)	9.78 ± 4.50	7.35 ± 3.99	10.54 ± 4.40	0.003
DC grade				0.006
Primary	67(72.8%)	11(50.0%)	56(80.0%)	
Secondary	25(27.2%)	11(50.0%)	14(20.0%)	
DC types				0.485
Unilateral	82(89.1%)	19(86.4%)	63(90.0%)	
Bilateral	8(8.7%)	2(9.1%)	6(8.6%)	
Bifrontal	2(2.2%)	1(4.5%)	1(1.4%)	
Operation time (min)	283.3 ± 114.4	342.2 ± 183.3	264.8 ± 74.6%	0.03
Intraoperative blood loss (ml)	1219.0 ± 744.9	1513.6 ± 768.0	1126.4 ± 718.2	0.025
Postoperative tracheotomy				0.036
With	58(63%)	18(81.8%)	40(57.1%)	
Without	34(37%)	4(18.2%)	30(42.9%)	
Subdural hygroma				0.022
With	43(46.7%)	15(68.2%)	28(40.0%)	
Ipsilateral	27(29.3%)	8(36.4%)	19(27.1%)	
Contralateral	10(10.9%)	5(22.7%)	5(7.1%)	
Bilateral	6(6.5%)	2(9.1%)	4(5.7%)	
Without	49(53.3%)	7(31.8%)	42(60.0%)	
Longitudinal fissure hygroma				0.004
With	21(22.8%)	10(45.5%)	11(15.7%)	
Without	71(77.2%)	12(54.5%)	59(84.3%)	
Ventricular dilatation	44	22(50%)	22(50%)	0.000
Postoperative large-area cerebral ischemia and infarction				0.020
With	35(38%)	13(59.1%)	22(31.4%)	
Without	57(62.0%)	9(40.9%)	48(68.4%)	

* The values are the number of patients (%), unless otherwise stated. The mean value is expressed as mean ± standard deviation.

**Table-II:** The relationship of subdural hygroma and longitudinal fissure hygroma with hydrocephalus in 92 patients with TBI undergoing DC.

Variables	Total cases*	PTH group*	Non-PTH group*
Subdural hygroma	43(11.0 ± 5.3 days)	15(13.0 ± 7.3 days)	28(10.0 ± 3.6 days)
Interhemispheric hygroma	21(16.4 ± 8.6 days)	10(18.7 ± 9.9 days)	11(14.3 ± 6.9 days)

* The value is the number of patients (days from DC when variable factors are found). The mean value is expressed as mean ± standard deviation.

### Univariate and multivariate analyses of hydrocephalus:

Table-III shows the univariate and multivariate analysis results of PTH. According to the univariate analysis results, statistically significant differences were found in the following variables, including DC grade (p=0.006), midline shift (p=0.004), operation time (p=0.005), intraoperative blood loss (p=0.032), tracheotomy (p=0.036), subdural hygroma (p=0.022), interhemispheric hygroma (p=0.004), ventricular dilatation (p=0.000), as well as large-area cerebral ischemia and infarction (p=0.002). Further multivariate analysis was conducted by involving all risk factors related to PTH formation with the elimination of confounding effects. It was found that subdural hygroma (OR=3.392 [95% CI=1.259-9.137]; p=0.016) was an independent risk factor for PTH formation after DC. The accuracy of subdural hygroma predicting PTH was estimated to be 88.0%.

**Table-III:** Univariate and multivariate analysis results of PTH formation.

Variables	Univariate analysis		Multivariate analysis	
	OR (95% CI)	P value	OR (95% CI)	P value
DC classification	4.000(1.422-11.098)	0.006	5.083(0.870-29.686)	0.071
Midline shift	0.838(0.740-0.948)	0.004	0.860(0.683-1.083)	0.200
Operation time	1.006(1.001-1.011)	0.005	1.007(0.997-1.017)	0.150
Intraoperative blood loss	1.001(1.000-1.001)	0.032	1.000(0.999-1.001)	0.635
Tracheotomy	3.375(1.035-11.009)	0.036	0.731(0.051-10.440)	0.817
Subdural hygroma	1.768(1.065-2.934)	0.022	3.392(1.259-9.137)	0.016
Longitudinal fissure hygroma	4.470(1.552-12.871)	0.004	0.769(0.132-4.473)	0.769
Ventricular dilatation	2.000(1.488-2.688)	0.000	2525051188	0.997
Large-area cerebral ischemia and infarction	3.152(1.173-8.468)	0.020	1.504(0.225-10.075)	0.674

### Management of PTH:

Of the 22 cases of PTH, 10 cases (45.5%) underwent CP, six cases (27.3%) received ventriculo-peritoneal shunt (VPS), nine cases (40.9%) were treated by lumbar cistern continuous drainage, and two cases (9.1%) were provided with lateral external ventricular drain (LEVD). Compared with non-operation group (n=12) with lumbar puncture and/or lumbar cistern drainage and/or LEVD, the curative effect was significantly better in the operation group with CP and/or VPS (p=0.029).

## DISCUSSION

The results of this study showed that the formation time of subdural hygroma was 11.0 ± 5.3 days, most of which were on the decompression side, some of which were on the opposite side. According to the report by Ban et al.^12^ The formation time of subdural hygroma was 10.8 ± 5.2 days after DC, which might occur on the decompression side or the opposite side, which supported the conclusion of this study. The incidence rate of PTH is high in TBI patients after DC,^13^ which seriously affects the prognosis of patients. Early prediction, early diagnosis and clinical precise treatment of PTH are beneficial to the prognosis of patients.^14^ In general, subdural hygroma begins to form within one week and peaks 3-4 weeks after operation.^15^

Furthermore, the occurrence of subdural hygroma suggests disordered CSF dynamics. Most cases have no obvious symptoms and dissipate voluntarily, while some cases may have the worsening progress of space occupying effect, and develop into PTH.^16^,^17^ In this study, multivariate analysis revealed that subdural hygroma was an independent risk factor for the formation of PTH, especially for the contralateral type, of which one half developed into PTH, which was consistent with these reported by Ki HJ et al.^18^ In case of persistent progression and aggravation of subdural hygroma after DC, much attention should be paid to the formation of PTH, which may be a sign of hydrocephalus.

PTH may develop in up to half of the patients with TBI who have interhemispheric hygroma after DC, suggesting that interhemispheric hygroma is a risk factor of PTH formation.^8^,^18^ As discovered by the present study, there was a high incidence of PTH formation in patients with interhemispheric hygroma. Furthermore, according to the results of univariate analysis, interhemispheric hygroma and subdural hygroma were risk factors of PTH after DC. The results of a meta-analysis reported by Victor M Lu et al support the conclusions of this study.^19^ Although the confounding effect of interhemispheric hygroma was ruled out by multivariate analysis, 47.6% of patients with interhemispheric hygroma formed PTH in clinical practice.

Furthermore, in this study, it was found that subdural and interhemispheric hygromas formed earlier than that of PTH, which was in accordance with multiple previous reports.^17^,^20^ In PTH group, six patients (27.3%) had concurrent formation of subdural hygroma and interhemispheric hygroma; while 83.3% of the patients formed subdural hygroma firstly, then interhemispheric hygroma, and finally developed into hydrocephalus. It in turn also suggests that the progressive aggravation of subdural hygroma and/or interhemispheric hygroma may play a predictive role in the formation of PTH.

Treatment of PTH aims to improve the progressive pathological state into the static state.^21^ Stiver et al.^22^ proposed that there was only a need for CP when PTH appeared after DC in some TBI patients, which could restore and normalize the dynamic function of CSF. In this study, six cases of PTH combined with skull defect were treated with CP first, among which two cases with cerebrospinal fluid circulation disorder were relieved by CP and had good prognosis. Heo J et al.^23^ reported that simultaneous CP and VPS increased the risk of postoperative complications and infection. In our study, four patients with significant expansion of decompression window were treated with VPS and then CP, with pressure-adjustable shunt valve applied in both procedures. For acute progressive PTH, VPS is not absolutely contraindicated. The most suitable valve pressure is recommended to be determined through scientific pressure regulation, and then CP can be performed timely according to the condition. In a word, individualized precise treatment should be applied for the treatment of PTH combined with skull defect according to the specific condition of patients. The conclusion of this study provides a new clinical basis for the occurrence and development, early diagnosis, risk prediction and precise treatment of PTH.

### Limitations

The present study still has the following limitations. This study was designed as a retrospective cross-sectional analysis, and it was difficult to avoid bias in the experimental design, which was inferior to prospective study in terms of completeness and accuracy. In addition, the sample size of PTH was relatively small, and not all patients with hydrocephalus received CP and/or VPS.

## CONCLUSIONS

PTH has a high incidence in TBI patients after DC, which seriously affects the survival and prognosis of patients. Subdural hygroma is an independent risk factor for PTH. Individualized precise treatment should be applied for the treatment of PTH combined with skull defect according to the specific condition of patients.

### Authors’ Contributions:

**ZG** and **HJ:** Designed this study, prepared this manuscript are responsible and accountable for the accuracy or integrity of the work.

**GZ** and **CX:** Collected and analyzed clinical data. Critical Review.

**ZZ:** Significantly revised this manuscript.

All authors have read and approved the final version.
